# RIG-I Mediates Innate Immune Response in Mouse Neurons Following Japanese Encephalitis Virus Infection

**DOI:** 10.1371/journal.pone.0021761

**Published:** 2011-06-30

**Authors:** Arshed Nazmi, Kallol Dutta, Anirban Basu

**Affiliations:** National Brain Research Centre, Manesar, Haryana, India; Global Viral Forecasting Initiative, United States of America

## Abstract

**Background:**

Neuroinflammation associated with Japanese encephalitis (JE) is mainly due to the activation of glial cells with subsequent release of proinflammatory mediators from them. The recognition of viral RNA, in part, by the pattern recognition receptor retinoic acid-inducible gene I (RIG-I) has been indicated to have a role in such processes. Even though neurons are also known to express this receptor, its role after JE virus (JEV) infections is yet to be elucidated.

**Methodology/Principal Findings:**

Upon infecting murine neuroblastoma cells and primary cortical neurons with JEV the expression profile of key proinflammatory cyto/chemokines were analyzed by qRT-PCR and bead array, both before and after ablation of RIG-I. Immunoblotting was performed to evaluate the levels of key molecules downstream to RIG-I leading to production of proinflammatory mediators. Changes in the intracellular viral antigen expression were confirmed by intracellular staining and immunoblotting. JEV infection induced neuronal expression of IL-6, IL-12p70, MCP-1, IP-10 and TNF-α in a time-dependent manner, which showed significant reduction upon RIG-I ablation. Molecules downstream to RIG-I showed significant changes upon JEV-infection, that were modulated following RIG-I ablation. Ablation of RIG-I in neurons also increased their susceptibility to JEV.

**Conclusions/Significance:**

In this study we propose that neurons are one of the potential sources of proinflammatory cyto/chemokines in JEV-infected brain that are produced via RIG-I dependent pathways. Ablation of RIG-I in neurons leads to increased viral load and reduced release of the cyto/chemokines.

## Introduction

Japanese encephalitis (JE) is the cause of recurrent epidemic viral encephalitis in south-east Asia, with around 30,000–50,000 cases reported every year resulting in 10,000 to 15,000 deaths [1]. The JE virus (JEV) is a positive sense single stranded (ss) RNA virus, with approximately 11 kb genome. JEV is a neuroptropic virus that causes extensive neuronal death, which is reportedly mediated by activating the tumor necrosis factor receptor (TNFR)-1 complex [2]. One of the hallmarks of JEV infection is activation of microglia which is associated with increased cell-proliferation and bioactive/inflammatory mediator production associated with distinct morphological changes. Although, certain activated glial-derived factors contribute to tissue repair, the interactive cross-talk between neuronal injury and microglial activation often determine the neuropathological outcome in JEV infection. The functional manifestations of such activated state are increased production of proinflammatory mediators like cyto/chemokines, reactive oxygen and nitrogen species by microglia. Increased release of proinflammatory mediators like tumor necrosis factor (TNF)-α, IL-1β, IL-6, MCP-1 and RANTES have been observed from microglia activated by JEV infection [3], [4], [5]. This release of cyto/chemokines represent the innate immune response against flaviviral infection of brain which in turn, is known to be mediated by the pattern recognition receptors (PRRs) such as toll like receptors (TLRs) and RIG-I–like receptors (RLRs) [6].

RIG-I, a type of RLR, recognizes double-stranded (ds) RNA and uncapped 5′ triphosphate RNA molecules, which are found in the genome of many RNA viruses or produced during viral replication. Interaction of viral RNA with RIG-I triggers several intracellular signaling pathways leading to activation of host cell cytokine gene expression and enhancement of other cellular functions. The pathway leading to activation of nuclear factor κB (NFκB) is activated by most, if not all, PRRs. NFκB plays a central role in driving the expression of proinflammatory mediators [7]. RIG-I has also been shown to activate p38MAPK, leading to secretion of CXCL-10, IL-12 and type-1 interferon in bone marrow-derived dendritic cells following viral infection [8].

Although microglia and astrocytes are classically believed to be the predominant source of these mediators in the brain, neurons can also express these factors in disease settings [9], [10]. JEV tropism for neurons is a well established fact. Hence, it would be plausible to assume that the neuronal PRRs play a certain role in mediating the ultimate fate of the infected neurons. It has been reported in earlier studies that JEV is recognized by RIG-I in various cells of myeloid and non-immune lineage [11]; but whether this is also true in neurons, is unknown. In recent years, the ‘immune unresponsive’ status of the CNS has been challenged by several studies. Apart from the glial cells, neurons have also been implicated to participate in innate immune defense against invading pathogens. The present investigation aims to determine whether viral recognition is RIG-I-dependent in neurons and also to evaluate role of the pathways downstream to RIG-I, in induction of proinflammatory mediators from JEV-infected mouse neurons.

## Methods

### Ethics statement

All animal experiments were approved by the Institutional Animal and Ethics Committee of National Brain Research Centre (approval nos. NBRC/IAEC/2007/36 and NBRC/IAEC/2008/41). Animals were handled in strict accordance with good animal practice as defined by Committee for the Purpose of Control and Supervision of Experiments on Animals (CPCSEA), Ministry of Environment and Forestry, Government of India.

### Virus and cells

The GP78 strain of JEV was propagated in suckling BALB/c mice and their brains were harvested when pathological symptoms were observed. Virus titrations were conducted and quantified as described earlier [12].

Mouse neuroblastoma Neuro2a (N2a) cells were obtained from National Centre for Cell Science, Pune, India. Cells were grown at 37°C in Dulbecco's modified Eagle medium (DMEM) supplemented with 3.7% sodium bi-carbonate (NaHCO_3_), 10% fetal bovine serum and penicillin/streptomycin. All the reagents related to cell culture were obtained from Sigma (St. Louis, USA), unless otherwise stated.

Cortical neurons were cultured following published protocol [13]. Briefly, cortices of P2 BALB/c mouse pups were dissected aseptically in calcium-magnesium-free (CMF)-Tyrode solution following decapitation. The meninges were removed, tissue were chopped into smaller pieces and collected in CMF-Tyrode. These were treated with trypsin DNAse and then dissociated in the same solution by triturating to make a single cell suspension, pelleted and resuspended in Neurobasal media. Neurobasal media was supplemented with L-glutamine (2 mM), 30% glucose, 5% fetal calf serum, 10% horse serum and penicillin–streptomycin. Cells were plated at a density of 5×10^3^ cells/cm^2^ onto poly-D-lysine-coated Labtek chamber slides and 96-well plate (Nunc, Roskilde, Denmark). After 48 h of incubation at 37°C, the serum containing medium was removed. Cells were incubated with serum free media for 4 h with antibiotics alone. For experimental treatments, the resting medium was exchanged for DMEM with N2 and B27 supplements, 25 mM KCl and antibiotics. Arabinoside (2×10^−5^ M) was used for the inhibition of astrocyte multiplication.

### Animal treatment

Five to six weeks old BALB/c mice of either sex were randomly distributed into 4 groups- Sham, JEV-infected, JEV-infected and treated with Vivo-morpholino against viral 3′ conserved region (JEV+3′ MO) and JEV-infected and treated with scrambled Vivo-Morpholino (JEV+S-MO). All Vivo-Morpholinos were commercially procured from Gene Tools LLC, (Philomath, OR, USA). Animals belonging to all groups except Sham were infected with 3×10^5^ plaque forming units (pfu) of JEV and that day was considered as day zero. Animals in Sham group received equal volume of filtered minimum essential media. Starting from 3 h post infection on day zero, 100 µg of S-MO (5′-ACTCCATCGTTCAGCCTCTGA-3′), and 3′ MO (5′-TCCCAGGTGTCAATATG CTCTT-3′), diluted in 0.1 mL of sterile 1× PBS (corresponding to 5 mg/kg body weight), were administered to animals belonging to JEV+S-MO and JEV+3′ MO groups respectively, once per day, for 5 consecutive days. Animals belonging to the Sham-treatment group received equal volumes of sterile 1× PBS [14]. JEV-infected and JEV+S-MO animals succumbed by 8^th^–9^th^ day post infection. The animals belonging to the Sham, and JEV+3′ MO groups were also sacrificed at same time point. Brain was excised out after repeated transcardial perfusion with ice-cold 1× PBS and were processed either for immunohistochemical staining or protein isolation.

### Infection and treatment schedule of neuronal cells

Cortical neurons were cultured in chamber slides in serum containing media, for 26 h. Cells were then either mock-infected or infected with JEV (2.3×10^8^ pfu/mL) at multiplicity of infection (MOI) of 5 for 1½ h. Cells were then washed twice with sterile 1× PBS to remove non-internalized virus and were incubated for 24 h in serum free media.

N2a were plated and cultured in serum containing media until 70–80% confluency. Cells were transfected with either morpholino directed against RIG-I (r-MO; 5′-AATTTCTTGGTTTCAATGGGC-3′) or a scrambled morpholino (Sc-MO; 5′-GATAATTCTGGTTTTAAATTC-3′), at 10 µM concentrations along with specialized delivery moiety (endoporter) and incubated for 2 h at 37°C. These morpholinos and the endoporter were also procured from Gene Tools LLC, (Philomath, OR, USA). Post incubation, cells were infected with JEV as described above and were then re-incubated with fresh morpholino-containing media for 24 h. Mock-infected cells with and without morpholino treatments served as controls; only JEV-infected cells, without morpholino treatment served as positive control.

### Determination of cell viability

N2a was plated onto separate 96-well plates in triplicate followed by RIG-I ablation and JEV infection. Cell viability was assessed using CellTiter 96 AQueous One Solution Cell Proliferation Assay kit (Promega, USA) as described earlier [13]. 24 h post infection, 20 µL of MTS solution was added in each well. After 4 h incubation in dark, the absorbance, reflecting the reduction of MTS by viable cells was determined at 490 nm. Values were expressed as a percentage relative to those obtained in mock-infected cells.

### Reactive oxygen species (ROS) generation assay

The level of intracellular ROS produced from different treatment groups were measured by the cell permeable, non-polar, H_2_O_2_-sensitive probe 5(and 6)-chlromethyl-20,70-dichlorodihydrofluoresceindiacetate (CM-H2DCFDA; Sigma, USA) as described earlier [13] with certain modifications. The mean fluorescence intensity was measured with the help of Varioskan Flash multimode reader (Thermo Electron, Finland) at excitation 500 nm and emission 530 nm. The fluorescence intensity of intracellular DCFDA is a linear indicator of the amount of H_2_O_2_ produced in the cells. The measured mean fluorescence intensity was then normalized to equal concentrations of protein in each sample.

### Cytokine bead array

Mouse cytokine bead array (CBA) kits (BD Biosciences, San Diego, CA) were used as per manufacturers' instructions to quantitatively measure cytokine levels in culture supernatants from all the treatment groups. Data were acquired using Cell Quest Pro Software in FACS Calibur and analyzed using BD CBA software (Becton Dickinson, San Diego, CA, USA) [13].

### Quantitative Real Time-PCR

Total cellular RNA from N2a treatment groups were extracted using Tri reagent (Sigma, USA). Random hexamer primers were used for cDNA synthesis using Advantage RT-PCR (Clontech, Mountain View, USA). cDNA were then subjected to mouse TLR signaling pathway PCR array (SA Biosciences, MD, USA) as per manufacturers instruction on a ABI Prism 7500 sequence detection system (Applied Biosystems, CA, USA). The real time PCR results were analyzed as per user manual guidelines and expressed as fold-changes over mock-infected.

### Immunostaining

Mouse cortical neuronal cells were cultured in 4-well chamber slides and were either mock-infected or infected with JEV as described above. After 24 h of incubation, the cells were fixed in 4% paraformaldehyde and blocked with 5% BSA solution. The cells were then incubated overnight at 4°C with primary antibodies against RIG-I and NeuN (1∶500; Chemicon, USA). Following incubation, the cells were washed and incubated with appropriate fluorochrome-conjugated secondary antibodies. After final washes cells were mounted with 4′-6-diamidino-2-phenylindole (DAPI, Vector laboratories Inc., CA, USA).

N2a were cultured in 8-well chamber slides and were treated with morpholinos followed by infection with JEV, as described under section infection and treatment schedule. The cells were immunostained using primary antibodies against phosphoNFκB (1∶250, Cell signaling, USA) and phosphoP38MAPK (1∶250, Cell signaling, USA) as described above. The slides were observed under a Zeiss Axioplan 2 Fluorescence microscope and images were captured using the AxioVision Rel 4.6 software (Carl Zeiss, CA, USA).

### Viral intracellular staining

N2a were plated in 60 mm culture plates and were treated as described above. After 24 h of incubation, cells were collected by washing with chilled sterile 1× PBS. After two washes with 1× PBS, cells were first fixed with BD cytofix solution (BD Biosciences) and permeabilized by resuspending in permeabilization buffer (BD Cytoperm plus; BD Biosciences). Cells were then washed twice in wash buffer (PBS containing 1% bovine serum albumin) and finally resuspended in wash buffer at 1×10^6^ cells per 100 µL. They were then treated with primary antibody (JEV Nakayama strain; Chemicon, USA; 1∶100 dilutions) for 30 min at room temperature. Cells were then washed and pelleted by centrifugation followed by incubation with FITC-conjugated secondary antibody for 30 min. After final wash, cells were resuspended in 400 µL FACS buffer and analyzed on a FACS Calibur. The percentage of population of JEV-positive cells was calculated after gating the populations on a Dot plot using Cell Quest Pro Software (BD Biosciences).

### Total cellular and nuclear protein extraction

N2a were seeded at a density of 5×10^6^ cell per 90-mm culture plates and were treated as described above. Mock-infected and treated N2a were collected using ice-cold 1× PBS. In order to obtain total cellular extracts, cells were lysed in buffer containing 1% Triton-X-100, 10 mM Tris-HCl (pH 8.0), 150 mM NaCl, 0.5% Nonidet P (NP-40), 1 mM EDTA, 0.2% EGTA, 0.2% sodium orthovanadate and protease inhibitor cocktail (Sigma, USA). To obtain nuclear extracts cells were washed twice with ice-cold PBS and pelleted. The cell pellet was resuspended in hypotonic buffer (10 mM HEPES, pH 8.0; 10 mM KCl; 5 mM dithiothreitol; 0.5 mM phenylmethylsulfonyl fluoride; 1 mM Na_3_VO_4_) and incubated on ice for 15 min. Then the cells were lysed by the addition of 0.5% IGEPAL CA 630 and vigorous vortexing for 30 s. The nuclei were pelleted by centrifugation at 12,000× *g* for 1 min at 4°C and resuspended in extraction buffer (20 mM HEPES, pH 8.0; 420 mM NaCl; 0.2 mM EDTA; 1 mM dithiothreitol; 0.5 mM phenylmethylsulfony fluoride; 1 mM Na_3_VO_4_). After 15 min on ice, lysates were centrifuged at 12,000× *g* for 10 min at 4°C [15]. Supernatants were collected and stored at −30°C. Protein levels were estimated by bicinchoninic assay (BCA) method.

### Immunoblotting

Western blot analysis was performed to study modulation in expression of proteins post treatment, from total cellular or nuclear extracts, using primary antibodies against JEV NS5 (a kind gift from Dr. Chun-Jung Chen, Taichung Veterans General Hospital, Taichung, Taiwan), Caspase 8, C23, FADD, TRAF-6, (kind gifts from Dr. Ellora Sen, NBRC), IPS-1 (Sigma, USA), RIG-I, phospho IKKα/β, IκBα, phospho and total NFκB, and phospho and total p38MAP kinase (Cell Signalling, USA) at 1∶1000 dilutions. After extensive washes with PBS–Tween, blots were incubated with appropriate peroxidase-conjugated secondary antibodies (Vector Laboratories, CA, USA). The blots were processed for development using chemiluminescence reagent (Millipore, USA). The images were captured and analyzed using Chemigenius, Bioimaging System (Syngene, Cambridge, UK). To determine equivalent loading of samples the blots were stripped and reprobed with anti-β-tubulin (Santa Cruz Biotechnology, CA, USA).

### Statistical analysis

Data were compared between groups using one-tailed Student's T Test. Statistical significance was set at p<0.05 for all analyses.

## Results

### JEV causes upregulation of RIG-I both in vivo and in vitro

**Figure 1:**
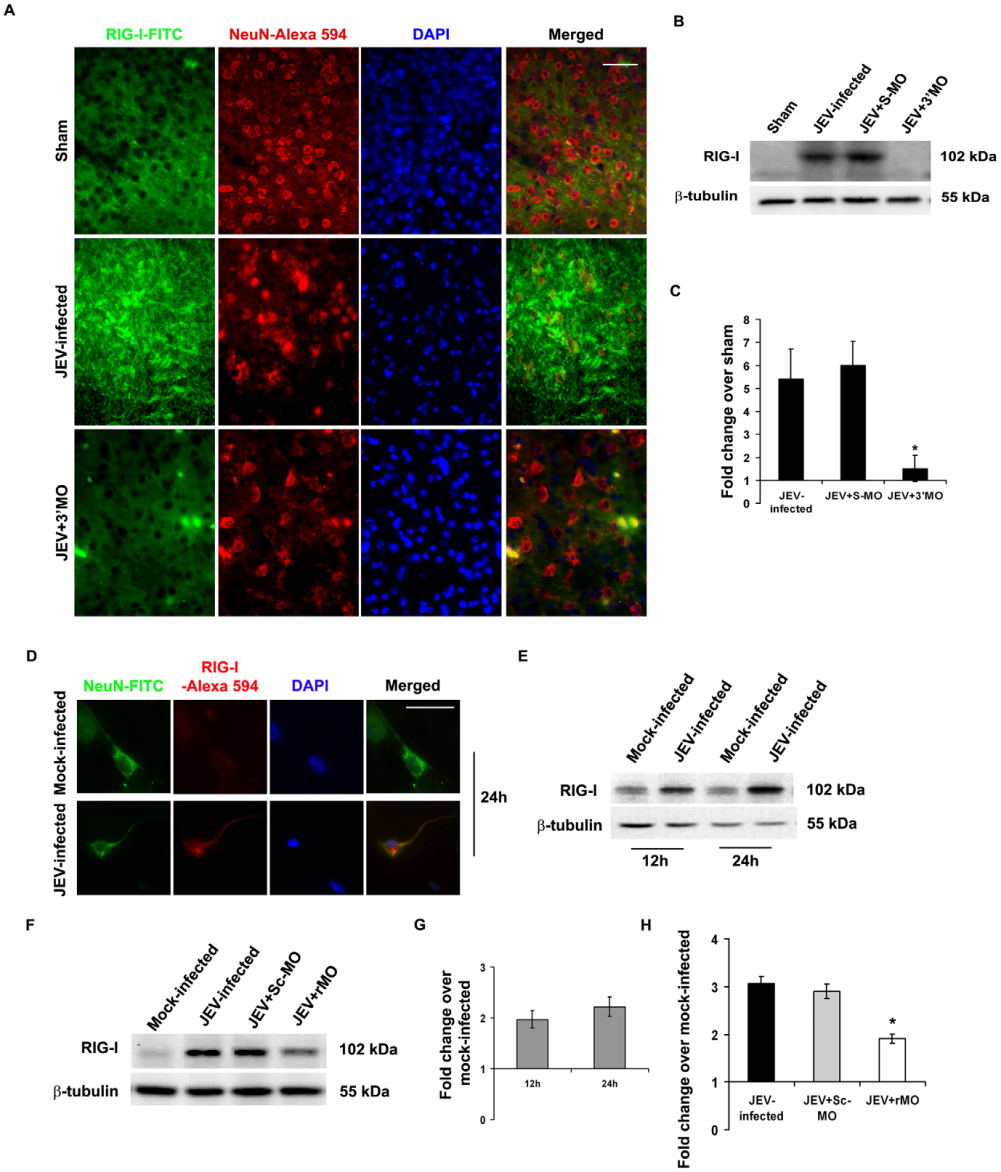
RIG-I expression in neurons following JEV-infection. Immunostaining of brain from JEV-infected animals showed increased expression of RIG-I, that was co-localized with neuron-specific marker NeuN, as compared to sham. Blocking of virus using 3′MO resulted in reduced detection of RIG-I in the brain. Magnification ×20; scale bar corresponds to 50 µ (A). Immunoblotting from whole brain lysates and their densitometric analysis showed increased (approximately 5-fold) expression of RIG-I in JEV-infected and JEV+S-MO groups. 3′MO treatment caused a significant decrease in RIG-I levels. (^*^p<0.05 when compared to JEV-infected) (B&C). Primary cortical neurons were cultured and stained for RIG-I following JEV infection. A prominent increase in RIG-I expression was observed when compared to mock-infected cells (D). N2a after JEV-infection showed increased expression of RIG-I both 12 and 24 h post-infection (E&G). Treatment with r-MO resulted in significant reduction in RIG-I expression; (^*^p<0.05 when compared to JEV-infected). Data is representative of 3 independent experiments (F&H).

Immunohistochemical analysis of brain sections of Sham, JEV-infected and JEV+3′ MO group of mice showed that there was prominent expression of RIG-I in JEV-infected group. However, viral inhibition by application of 3′ MO resulted in RIG-I expression that was comparable to Sham (*Fig. 1A*). Immunoblot analysis from whole brain lysates confirmed significant increase of RIG-I in JEV-infected or JEV+S-MO groups that was drastically reduced following treatment with 3′ MO (p<0.05) (*Fig. 1B&C*). RIG-I expression was also found to be enhanced following JEV infection in cultured primary cortical neurons (Fig. 1D). Immunoblotting from N2a cells post 12 h and 24 h of infection showed significant increase in RIG-I, when compared to mock-infected cells. However, there was no significant difference in RIG-I levels between the two time points (*Fig. 1E&G*). Application of rMO resulted in approximately 50% reduction in RIG-I level even after 24 h post JEV infection (p<0.05) (*Fig. 1F&H*).

### JEV infection causes modulations in expressions of molecules downstream to RIG-I leading to activation of p38MAPK and NFκB

**Figure 2:**
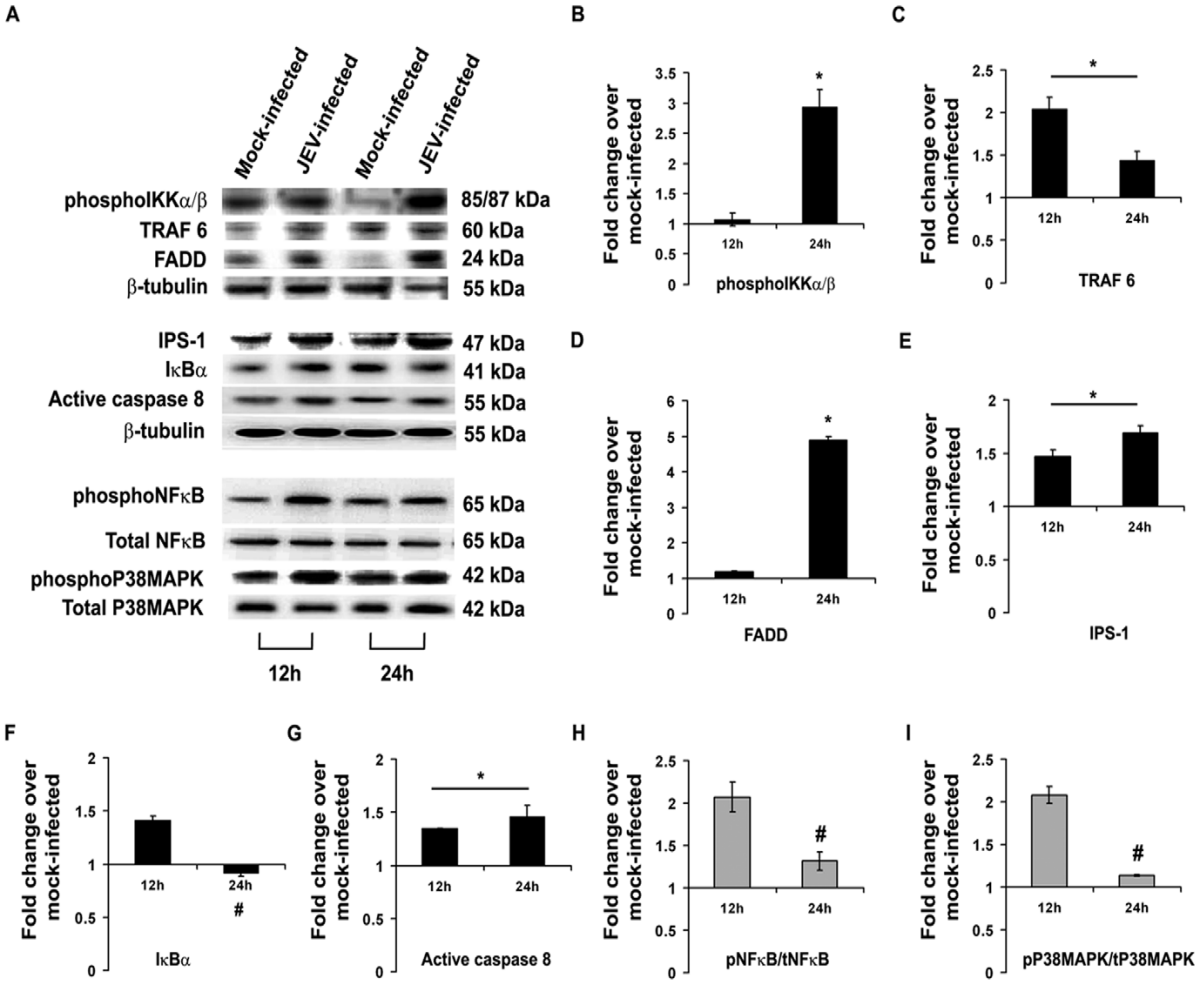
Temporal changes in the levels of key signaling molecules in neurons leading to activation of P38MAPK and NFκB, post infection with JEV. Immunoblotting for molecules downstream to RIG-I was performed from protein isolated from N2a, 12 and 24 h post infection with JEV. TRAF-6, IPS-1 and active Caspase8 levels in JEV-infected N2a showed significant increases both 12 and 24 h post-infection, but phospho IKKα/β and FADD levels were increased significantly only after 24 h post-infection when compared to mock-infected (A–E & G). The level of IκBα was found to be significantly lower in 24 h post-infected group when compared to 12 h post-infected group (A&F). PhosphoP38MAPK and phosphoNFκB levels were found to be considerably increased after 12 h post-infection as compared to mock-infected. But after 24 h their levels were significantly lesser than 12 h post infection groups (A, H & I). (^*^p<0.05 when compared to mock-infected; ^#^p<0.05 when compared to 12 h post-infected). Data is representative of 3 independent experiments.

**Figure 3:**
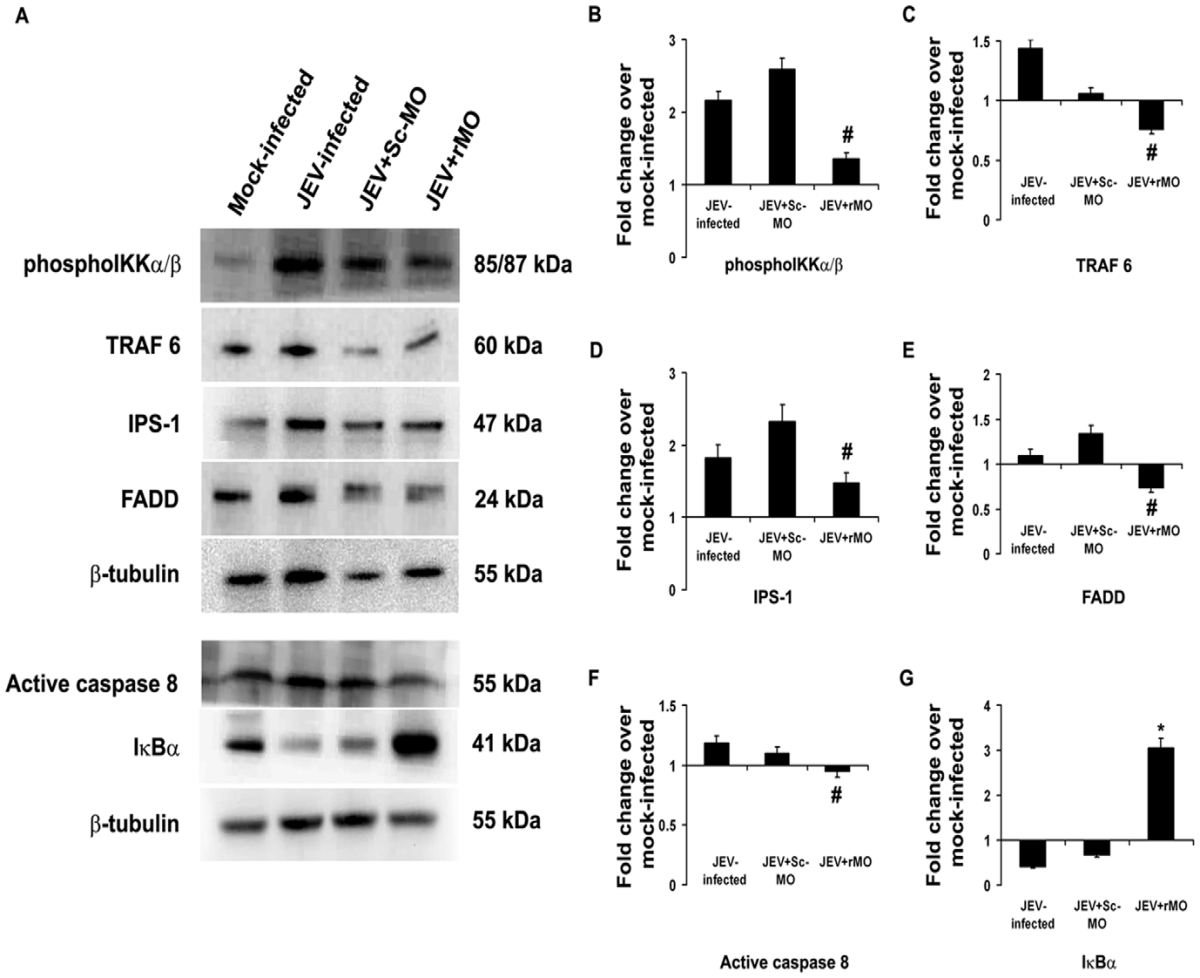
Ablation of neuronal RIG-I decreases the levels of key signaling molecules post JEV-infection. Following ablation of RIG-I and 24 h post infection with JEV, the levels of certain downstream elements of the RIG-I pathway were evaluated by immunoblotting protein isolated from N2a. PhosphoIKKαβ, TRAF-6, FADD, IPS-1 and active Caspase8 levels in JEV+rMO group showed significant decreases when compared to JEV-infected groups (A–F). The level of IκBαwas found to be significantly increased in JEV+rMO when compared to JEV-infected group (A & G) (^#^p<0.05 when compared to JEV-infected group, ^*^p<0.05 when compared to JEV-infected group). Data is representative of 3 independent experiments.

**Figure 4:**
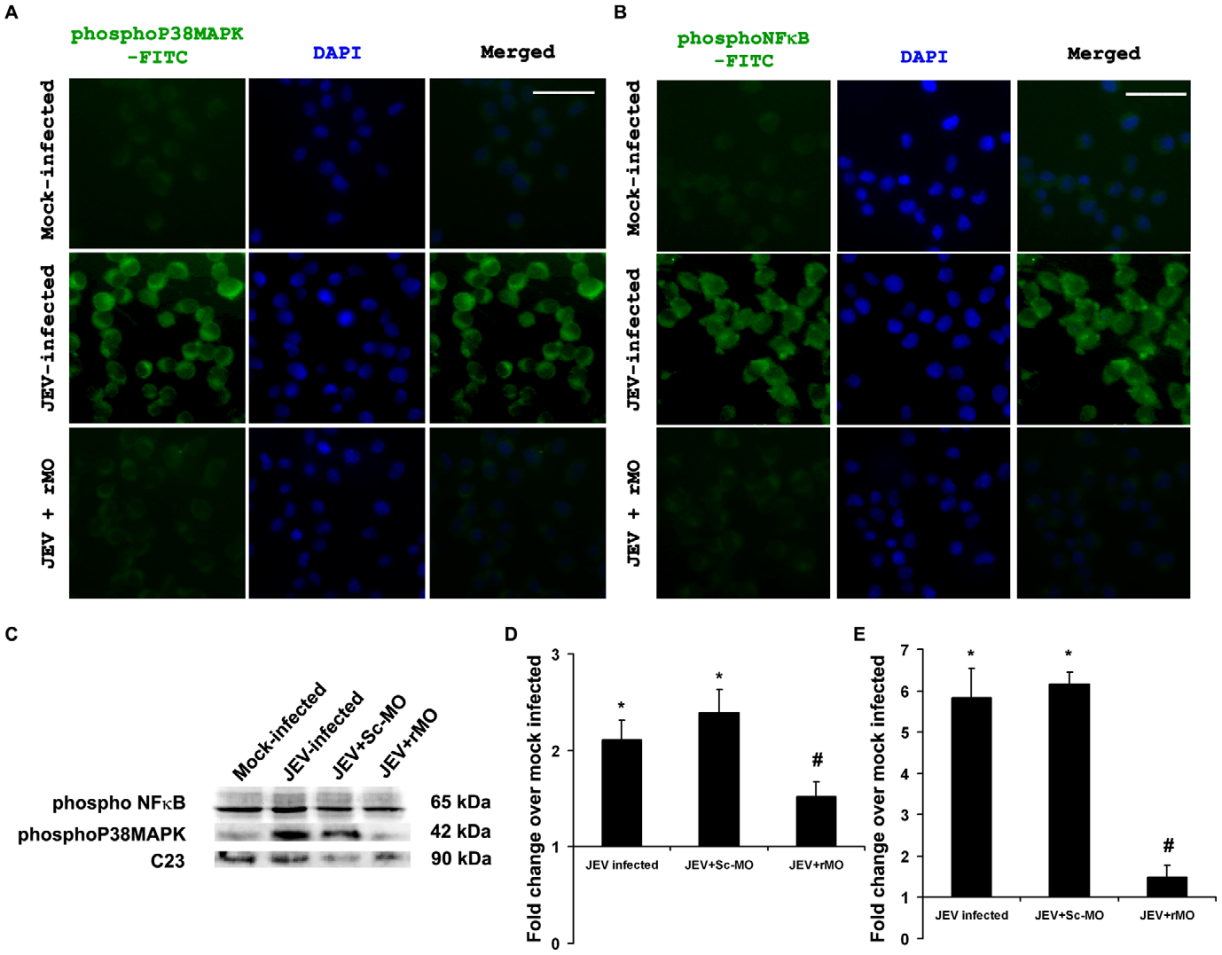
Decrease in levels of activated P38MAPK and NFκB in JEV-infected neurons post RIG-I ablation. Mock-infected, JEV-infected and JEV+rMO treated N2a were stained for the detection of pP38MAPK and pNFκB, 24 h post infection. Photomicrographs show decreased expression of either of them in JEV+rMO when compared to JEV-infected groups. Magnification ×20; scale bar corresponds to 50 µ (A&B) Immunoblotting for these molecules from nuclear extracts of all the groups of cells showed considerable decreases in JEV+rMO groups when compared to JEV-infected groups. The data were normalized to mouse nuclear protein C23 (C–E). (^*^p<0.05 when compared to mock-infected, ^#^p<0.05 when compared to JEV-infected). Data is representative of 3 independent experiments.

Post JEV infection, the level of molecules associated with RIG-I signaling was investigated by immunoblotting. It was observed that the levels of IPS-1, TRAF 6 and active Caspase 8 were significantly increased both after 12 and 24 h post infection, whereas the levels of phosphoIKKα/β and FADD were only increased after 24 h (p<0.05). IκBα, phosphoNFκB and phosphoP38MAPK levels were found to be decreased significantly (p<0.05) after 24 h post infection compared to 12 h post infection groups (*Fig. 2*). Following RIG-I inhibition, it was seen that phosphoIKKα/β, FADD, IPS-1, TRAF 6 and active Caspase 8 levels were significantly reduced 24 h post infection when compared to only JEV-infected or JEV+Sc-MO group (p<0.05) , while the level of IκBα was significantly increased (p<0.05) (*Fig. 3*). Treatment of mock-infected cells with only rMO did not result in any significant change in the expression levels of these molecules (*Fig. S1A–F*) which shows that the changes were due to the virus infection. Immunocytochemical staining revealed increased expressions of phosphorylated form of NFκB and P38MAPK was expressed in higher degree 24 h post infection when compared to mock-infected cells. However, following rMO treatment, their levels were found to be comparable to mock-infected cells even after 24 h of JEV infection (*Fig. 4A&B*). Immunoblotting from nuclear extracts of cells from all the treatment groups showed significantly increased levels of both phosphoNFκB and phosphoP38MAPK in JEV-infected and JEV+Sc-MO groups as compared to mock-infected (p<0.05). After RIG-I inhibition, their levels were found to be significantly decreased when compared to JEV-infected group (p<0.05) (*Fig. 4C–E*).

### Transcriptional alterations of certain signaling molecules associated with RIG-I following JEV infection

**Figure 5:**
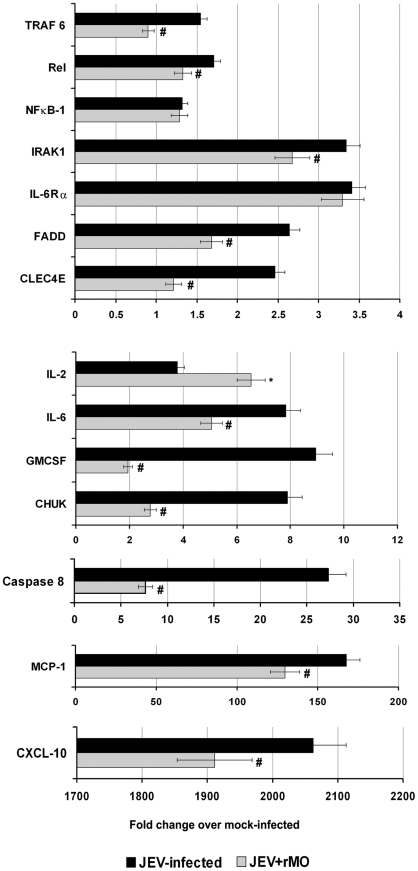
Gene expression profile of downstream pathways and target genes associated with RIG-I. N2a were mock-infected, JEV-infected or JEV-infected and treated with rMO, followed by incubation for 24 h. Total RNA was extracted for quantitative real time PCR (qRTPCR) array analysis. The data are normalized to mouse β-actin messenger RNA (mRNA) and are expressed as the relative fold increase over normalized RNA from mock infected cells. Data is representative of 2 independent experiments. (^*^p<0.05 when compared to JEV-infected, ^#^p<0.05 when compared to JEV-infected).

Quantitative real time PCR was carried out to determine the transcriptional changes in certain effectors and downstream target genes associated with RIG-I. Specifically, the expression of effector molecules such as Caspase 8, FADD, IRAK 1 and TRAF 6 were found to be significantly increased following JEV infection. Among the downstream target genes, the levels of MCP-1, CXCL-10, CHUK, GMCSF, IL-6, IL-2, CLEC4E, IL-6Rα, NFκB-1 and Rel were also found to be significantly increased. On ablating RIG-I with the application of rMO, it was found that there was significant decrease in all the studied effector molecules. Amongst the studied downstream molecules all, except NFκB-1, IL-6Rα and IL-2, showed significant decreases when compared to JEV-infected group (p<0.05). IL-2 level was found to be significantly increased after rMO treatment (p<0.05) (*Fig. 5*).

### Ablation of RIG-I abrogates secretion of proinflammatory cyto/chemokines from neurons following JEV infection

**Figure 6:**
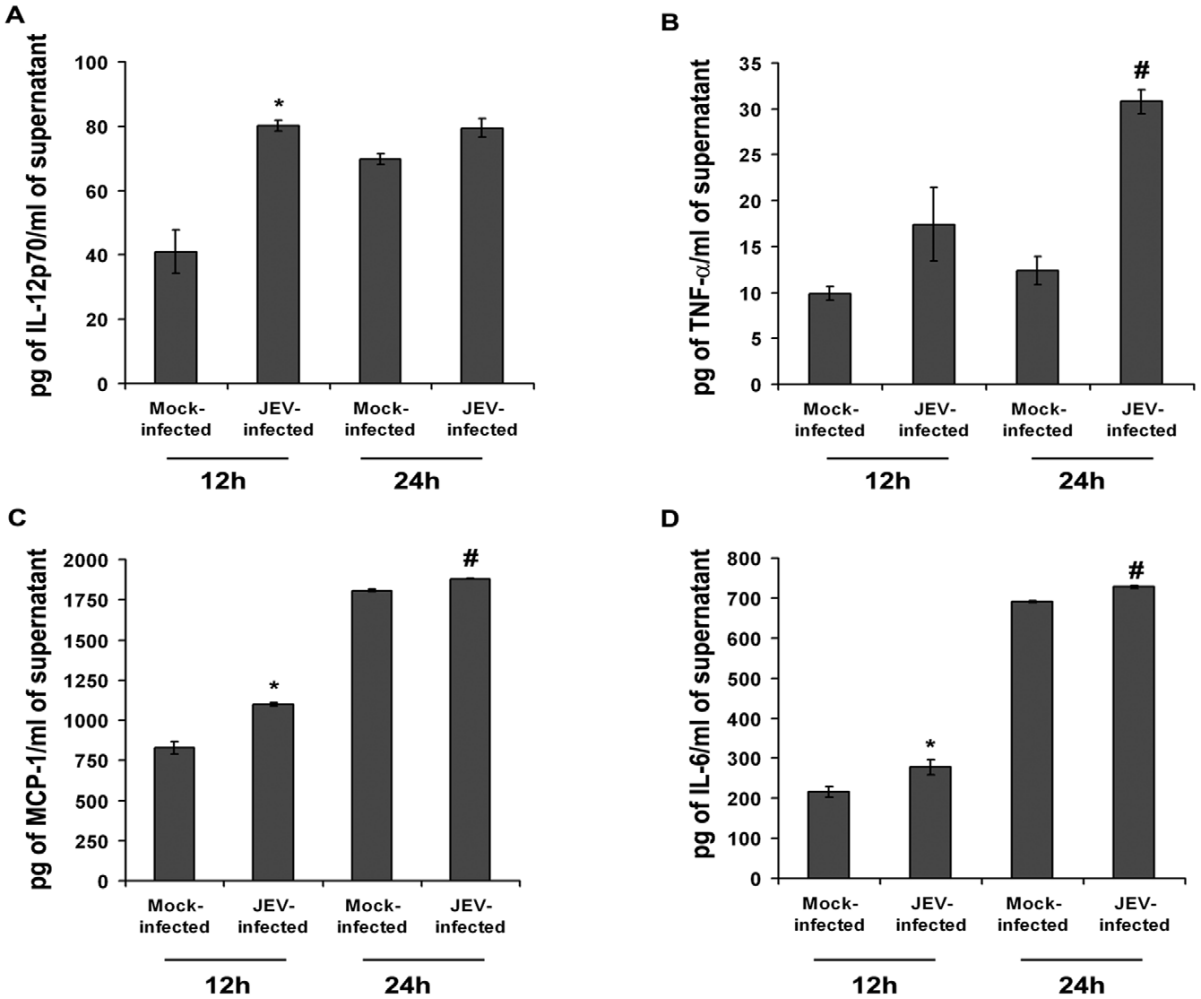
Neuronal proinflammatory cyto/chemokine profile following JEV infection at different time points. Cytometric bead array performed from culture supernatants obtained from JEV-infected N2a, 12 h and 24 h post infection showed that IL-12p70, TNF-α, IL-6 and MCP-1 levels were significant increased in comparison to respective time-matched mock-infected groups (A–D) (^*^p<0.05, when compared to mock-infected after 12 h, ^#^p<0.05, when compared to mock-infected after 24 h). Values are mean ± SD of 3 independent experiments.

**Figure 7:**
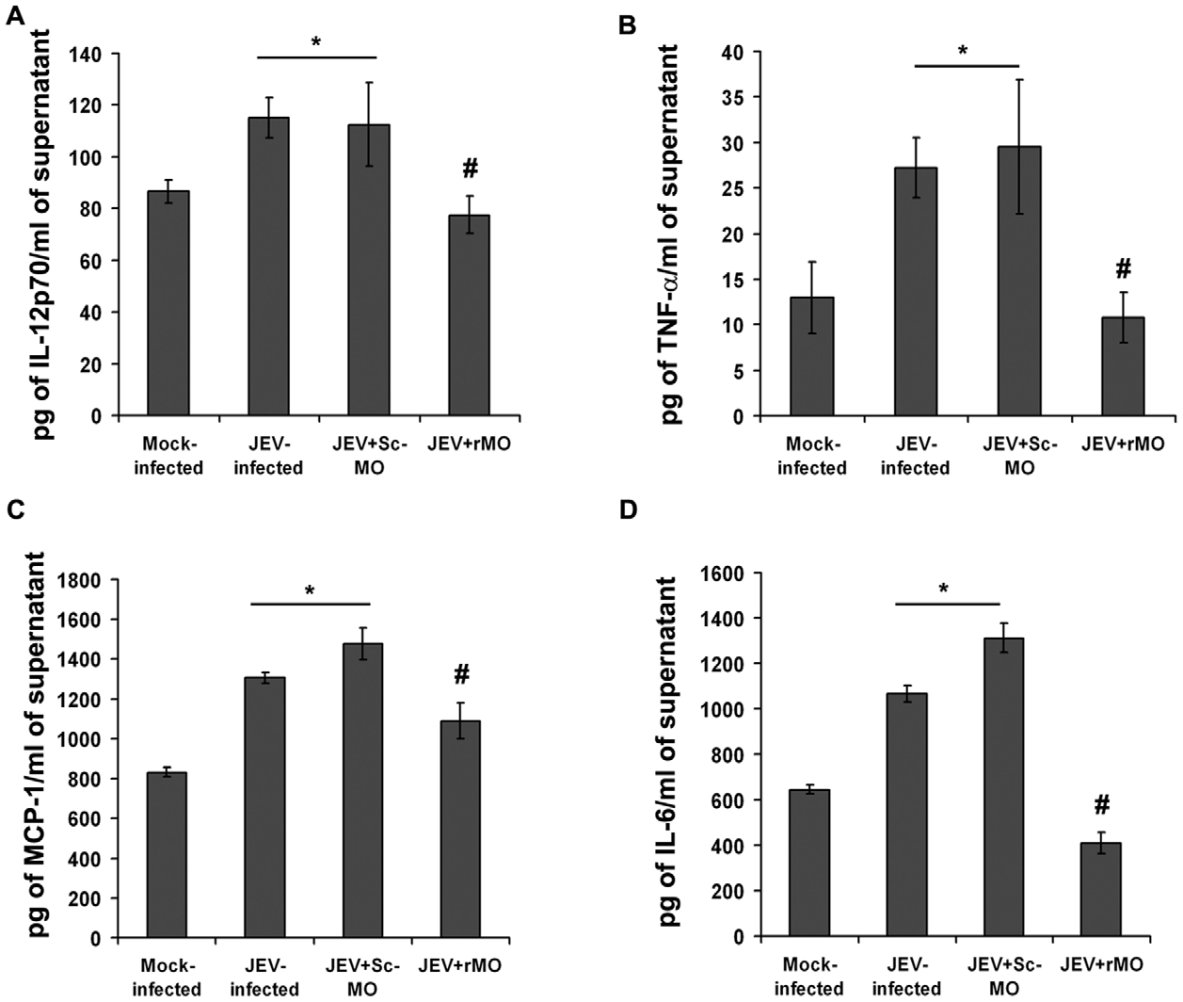
Comparison of proinflammatory cyto/chemokine profile following JEV infection pre- and post- ablation of RIG-I. Following JEV infection and treatment with either Sc-MO or rMO, the cyto/chemokine levels showed significant increases in supernatants from JEV-infected and JEV+Sc-MO groups. However, significant reductions were observed in their levels following RIG-I ablation, 24 h post-incubation (A–D). (^*^p<0.05, when compared to mock-infected, ^#^ p<0.05, when compared to JEV-infected). Values are mean ± SD of 3 independent experiments.

The release of proinflammatory cyto/chemokines viz. IL-12, TNF-α, MCP-1 and IL-6, were found to be significantly elevated both 12 and 24 h post JEV infection when compared to that from respective time-matched mock-infected cells (p<0.05) (*Fig. 6A–D*). However, ablation of RIG-I resulted in significant reduction in their levels 24 h post infection with JEV when compared to only JEV-infected or JEV+Sc-MO group (p<0.05) (*Fig. 7A–D*). rMO itself did not cause appreciable change in cytokine levels (*Fig. S1G–J*).

### RIG-I ablation results in increased viral load, decreased survivability and generation of oxidative stress in N2a

**Figure 8:**
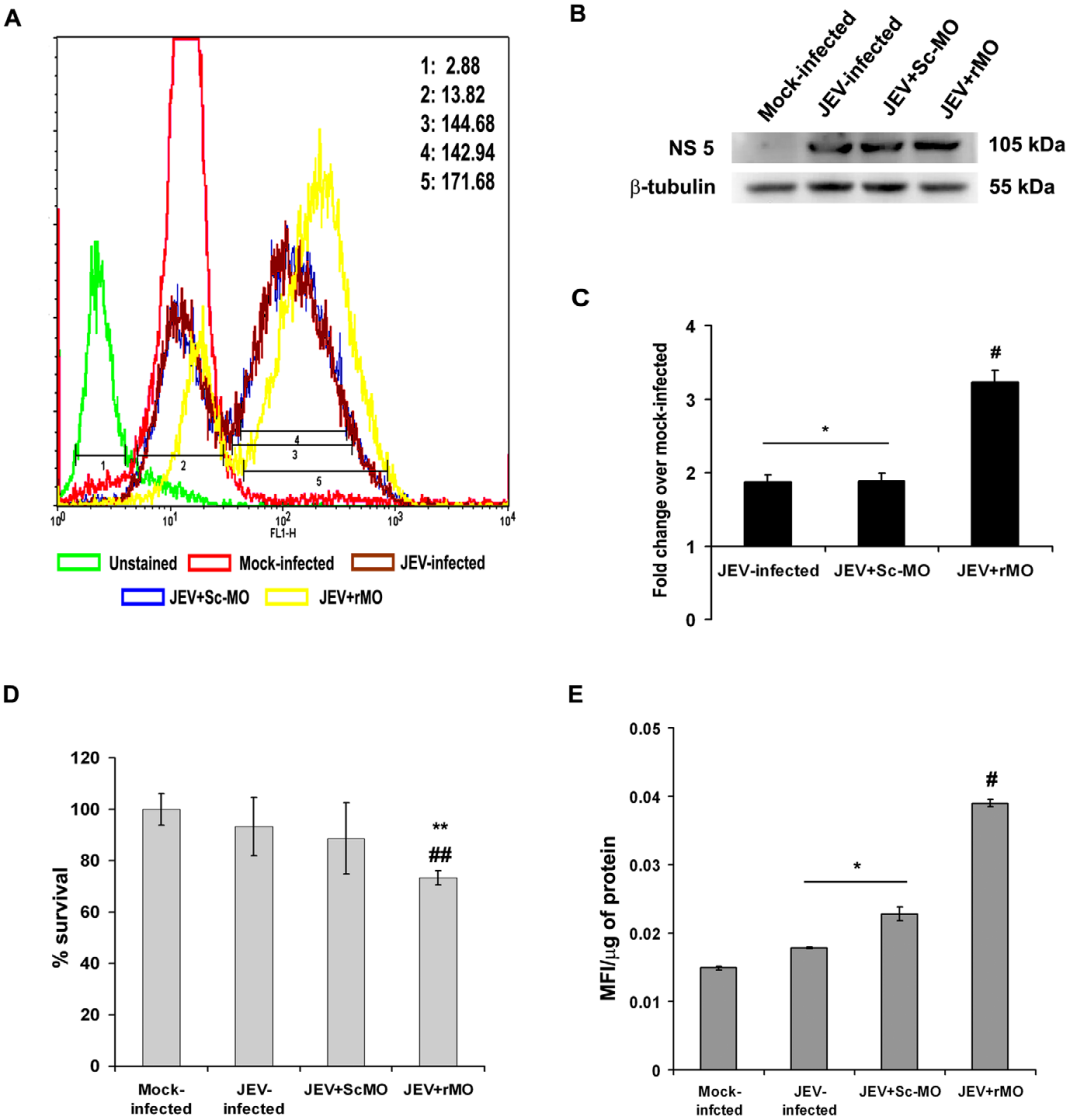
Increased oxidative stress, viral load and decreased survivability of N2a following RIG-I ablation. Flow cytometric analysis carried out from cells of all treatment groups to detect intracellular JEV antigen showed significant increase post RIG-I ablation when compared to only JEV-infected or JEV+ScMO groups. The mean fluorescence intensities obtained from each group are depicted in the figure. (1-Unstained, 2-Mock-infected; 3-JEV-infected, 4-JEV+Sc-MO, 5-JEV+rMO) (A). Immunoblot analysis to detect JEV NS-5 expression also showed enhancement in JEV+rMO when compared to either JEV-infected or JEV+ScMO groups (B&C). (^*^p<0.05, when compared to mock–infected, ^#^p<0.05 when compared to JEV-infected group). No statistically significant change in the viability of N2a post 24 h of JEV-infection was observed. However, RIG-I ablation resulted in significantly decreased viability compared to mock-infected as well as JEV-infected groups (D). (^**^p<0.05 when compared to mock-infected; ^##^p<0.05 when compared to JEV-infected). ROS production, a hallmark of JEV infection, showed significant increases in JEV-infected and JEV+Sc-MO groups when compared to mock-infected groups which on RIG-I ablation showed further increase even when compared to JEV-infected group (E). (^*^p<0.05 when compared to mock-infected; ^#^p<0.05 when compared to JEV-infected). Data is representative of 3 independent experiments.

Flow cytometric analysis showed increased presence of viral antigen after intracellular staining in JEV+rMO group when compared to JEV-infected or JEV+Sc-MO groups (*Fig. 8A*). Immunoblot analysis of viral NS5 also showed significant increase in JEV+rMO group when compared to JEV-infected or JEV+Sc-MO groups (p<0.05) (*Fig. 8B&C*). MTS assay performed on all treatment groups showed reduced survivability in JEV-infected or JEV+Sc-MO groups when compared to mock-infected, though the change was not statistically significant. However, in the JEV+rMO group survivability was significantly decreased when compared to mock-infected, JEV-infected or JEV+Sc-MO groups (p<0.05) (*Fig. 8D*). Generation of oxidative stress is a hallmark of JEV-infected cells. Here, we found that ROS levels were significantly increased in JEV-infected or JEV+Sc-MO groups when compared to mock-infected groups. However, in JEV+rMO group, the ROS level was found to be even higher than either JEV-infected or JEV+Sc-MO groups (p<0.05) (*Fig. 8E*).

## Discussion

Multiple cell types in the body have been reported to be capable of sensing flaviviral infections and initiating innate immune responses. Double stranded (ds)RNA, generated during infection as a replication intermediate for RNA viruses, serve as pathogen-associated molecular pattern that can be recognized by several PRRs. RIG-I, a cytoplasmic protein that has been shown to be a detector of dsRNA as well as ssRNA with 5′ triphosphates [16]. Recognition of flaviviruses through RIG-I has been reported [17] that results in type I interferon and proinflammatory cyto/chemokine production from cells of the myeloid lineage [18]. In vitro studies have also shown that cells of non-immune lineage primarily respond to intracellular accumulation of viral dsRNA via RIG-I [19]. Specifically, RIG-I has been shown to be essential in the identification of JEV for the production of type I interferon in Vero (epithelial) cells [20].

The innate immune responses of neurons following JEV infection is poorly understood as the CNS has for long, been considered as an ‘immune privileged’ organ. It has been reported that PRRs are expressed in neurons [21]. RIG-I is basally expressed in undetectable levels in uninfected neurons [22], though recent studies suggest that it plays a prominent role in mediating innate immune response, following viral infections [23]. In this study, prior to JEV infection, we found very low expression of RIG-I both in whole brain and in cultured neurons. JEV predominantly infects neurons, following which RIG-I was found to be expressed in significantly higher levels in brain. As after inhibiting the virus using an antisense molecule (3′ MO) no significant alteration in RIG-I expression was observed from non-infected brain samples, it can be safely assumed that JEV causes upregulation of this PRR in various CNS cell types, including neurons. Upon recognition of viral RNA, the caspase recruitment domain (CARD)-like motif of RIG-I gets activated and interacts with interferon promoter stimulator-1 (IPS-1) to relay the signal. The signal is divided at IPS-1, resulting in activation of NFκB and IRF-3 and -7 [7]. Post JEV infection, IPS-1 expression was found to be moderately increased that led to dramatic increase of the adaptor molecule FADD. Apart from its role in apoptotic process, FADD is also known to activate TNF receptor-associated factor 6 (TRAF 6), which was found to be increased following JEV infection in neurons. TRAF 6 activates IκB kinase α/β (IKKα/β) via Tak1 leading to phosphorylation of IκBα-NFκB complex. JEV infection leads to decreased level of IκBα, at later time point indicating its dissociation from the complex followed by proteolytic degradation. Since the removal of the inhibitory effect of IκBα, NFκB is activated and translocated into the nucleus to modulate the expression of proinflammatory cyto/chemokine genes. We found increased level of phospho NFκB in neuronal cells following JEV infection. Its level in nuclear extract of these cells was also increased. Activation of p38MAPK has been shown to be associated with the activation of RIG-I via TRAF 2-TAK 1 dependent pathway [8] in non-neuronal cells. Whether this also occurs in neurons was previously unknown. Here we have shown for the first time that following JEV infection, phospho p38MAPK levels are significantly increased in neurons as evident from immunocytochemical observations and also from immunoblot analysis of nuclear extracts obtained from infected cells. Both NFκB and p38MAPK are well known stimulators of proinflammatory cyto/chemokine gene expression, and their upregulation was concurrent with elevated secretions of IL-12, TNF-α, MCP-1 and IL-6 and increased expression of CXCL-10 (IP-10), GMCSF from infected neuronal cells. RIG-I inhibition carried out by transfecting cells with rMO resulted in downregulation of phosphoIKKαβ, TRAF 6, IPS-1, FADD, and active Caspase 8, except IκBα, which showed significant increase indicating its inhibitory effects on NFκB. Subsequently, it was observed that phospho NFκB and phospho p38MAPK levels were decreased that led to reduction in the levels of the proinflammatory mediators.

**Figure 9:**
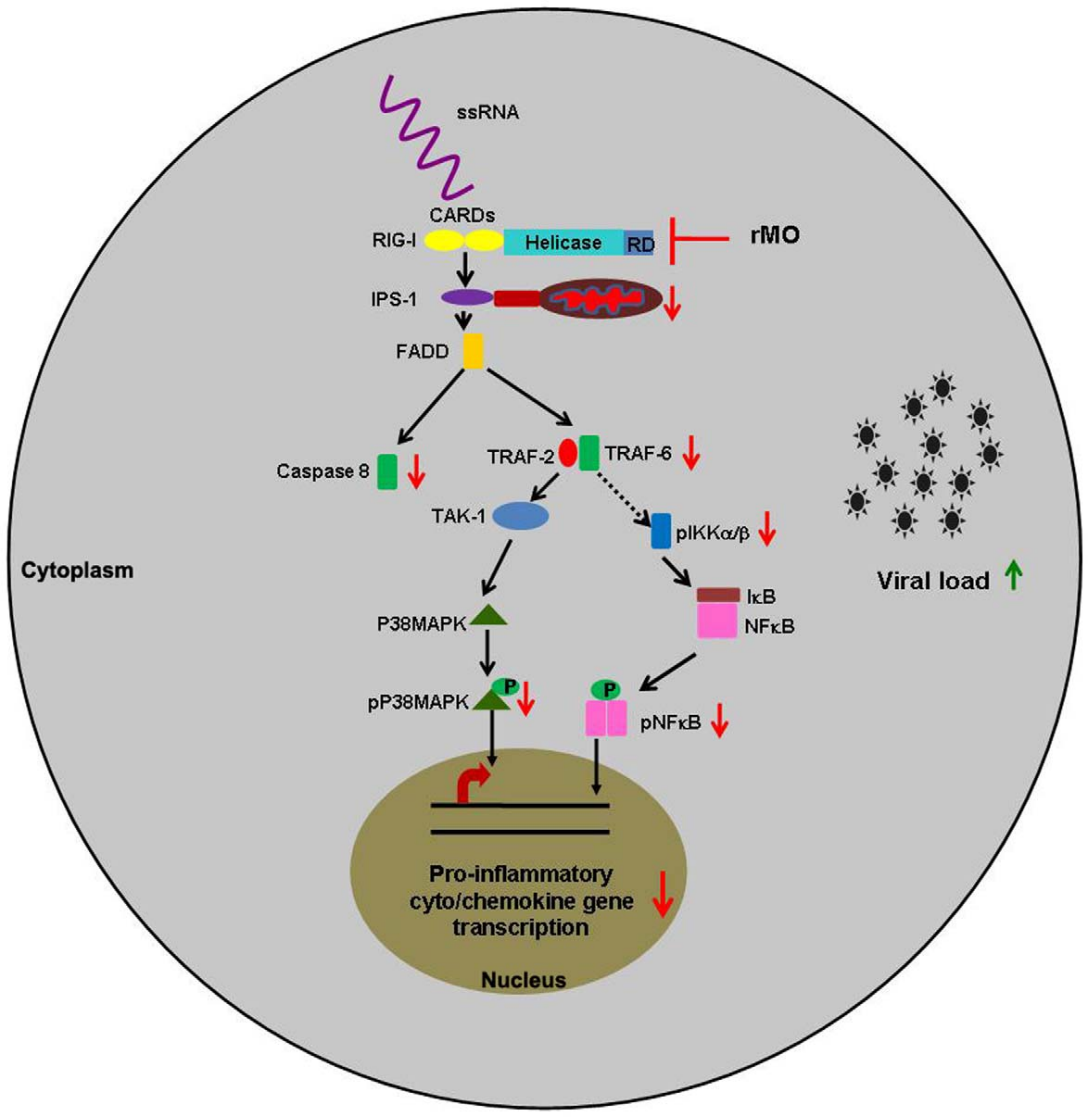
Schematic diagram showing RIG-I signaling pathway leading to production of proinflammatory cyto/chemokines from neurons. Detection of viral ss/dsRNA by RIG-I activates a cascade of changes that leads to activation of P38MAPK and NFκB. As a result of such activation, proinflammatory cyto/chemokine gene transcription is enhanced in neurons that ultimately contribute to the inflammatory milieu in the brain, following JEV infection. Ablation of RIG-I using antisense morpholino oligonucleotide results in negative modulation of this pathway. ↑-upregulation; ↓-downregulation; ⊢ -ablation.

An interesting observation was that neuronal survivability was negatively affected following RIG-I inhibition. JEV-infection results in development of oxidative stress and leads to apoptotic death of neuronal cell lines [24]. Ablation of RIG-I was found to be associated with decreased survivability and increased stress in the cells in comparison to only JEV infected cells. This was concomitant with increased intracellular viral load, as shown by immunoblot and flow cytometric analysis. This phenomenon may be due to the fact the antiviral innate immune response is subverted following RIG-I ablation and this observation merits further studies. Thus, it is clear from this study that viral sensing through RIG-I lead to modulations in the downstream pathway (*Fig. 9*) that cause the infected neurons to participate in the inflammatory milieu in CNS which ultimately leads to their own demise. However, the concerted role of other PRRs cannot be ruled out at this point of time. Since there are other sensors present in cells such as TLRs, PKR, MDA5 and MPYS, a co-receptor effect is probable, but not yet elucidated in case of JEV infection.

## Supporting Information

Figure S1 **The antisense rMO treatment of mock-infected cells does not result in modulation of downstream signaling molecules or proinflammatory cyto/chemokine release.** On application of rMO to mock-infected cells, the expression levels of TRAF6, IPS-1, active Caspase 8, IκBα and FADD were found not to be significantly different from that observed in only mock-infected cells (A–F). To check whether rMO induced proinflammatory cytochemokine release from cells, CBA was performed from culture supernatants. Results showed that there were no significant differences between the cyto/chemokine levels in mock-infected and mock-infected+rMO treated groups (G–J). Data is representative of 2 independent experiments.
(TIF)
