# Supplementary figures and images for: RIG-I Mediates Innate Immune Response in Mouse Neurons Following Japanese Encephalitis Virus Infection
Source: PLoS One. 2011 Jun 30;6(6):e21761. doi: 10.1371/journal.pone.0021761 (PMC3128083; doi:10.1371/journal.pone.0021761)

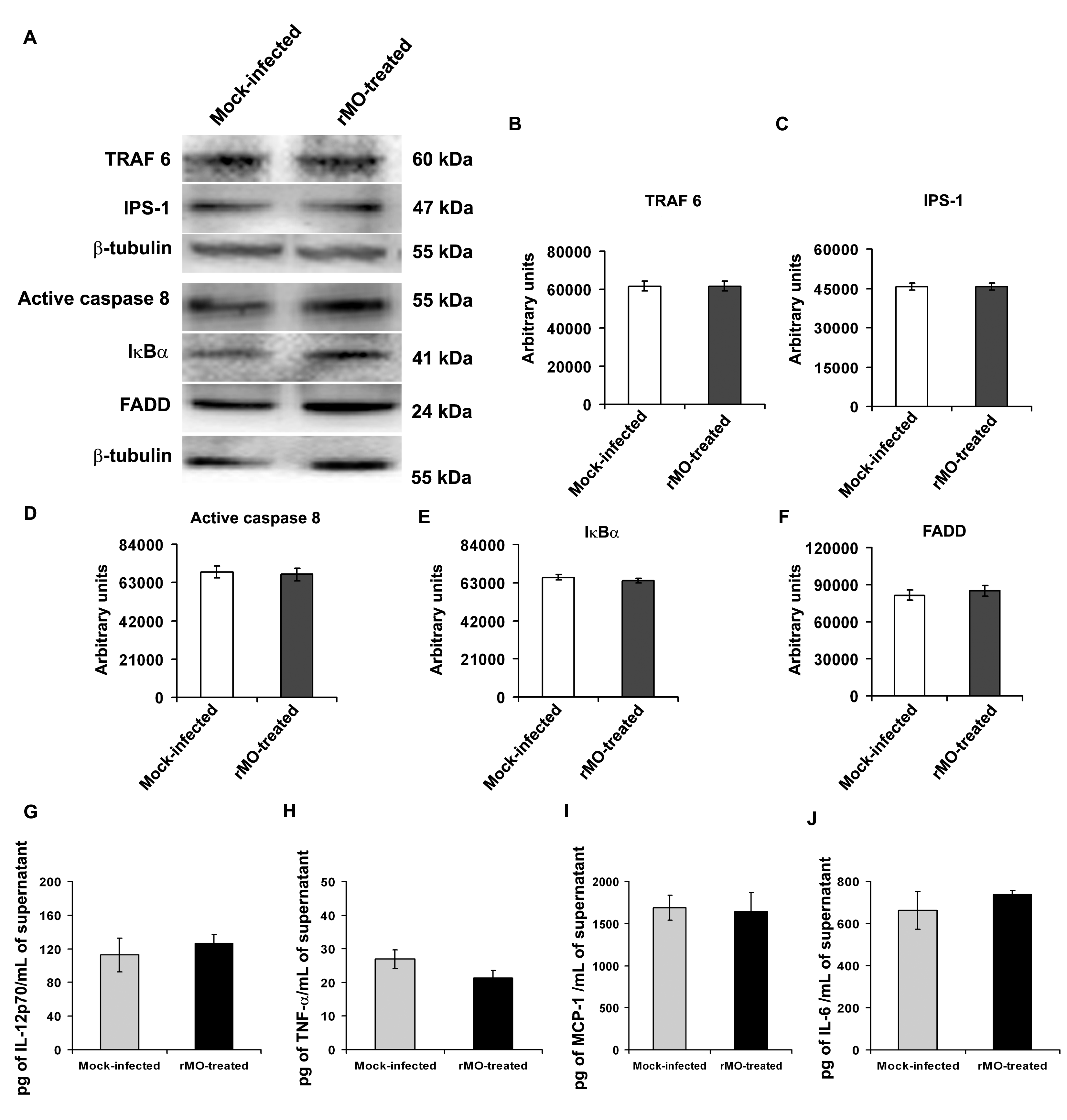

Supplement: Figure S1 — The antisense rMO treatment of mock-infected cells does not result in modulation of downstream signaling molecules or proinflammatory cyto/chemokine release. On application of rMO to mock-infected cells, the expression levels of TRAF6, IPS-1, active Caspase 8, IκBα and FADD were found not to be significantly different from that observed in only mock-infected cells (A–F). To check whether rMO induced proinflammatory cytochemokine release from cells, CBA was performed from culture supernatants. Results showed that there were no significant differences between the cyto/chemokine levels in mock-infected and mock-infected+rMO treated groups (G–J). Data is representative of 2 independent experiments. (TIF) [file pone.0021761.s001.tif]
